# Data set on trace metals in surface sediment and water from a sub-tropical estuarine system, Bay of Bengal, Bangladesh

**DOI:** 10.1016/j.dib.2020.105911

**Published:** 2020-06-23

**Authors:** M. Belal Hossain, Solaiman Bin Habib, Md. Solaiman Hossain, Y.N. Jolly, Abu Hena Mustafa Kamal, Mohd Hanafi Idris, Md. Refat Jahan Rakib

**Affiliations:** a Department of Fisheries and Marine Science, Noakhali Science and Technology University, Noakhali-3814, Bangladesh; b Department of Oceanography, School of Physical Sciences, Shahjalal University of Science and Technology, Sylhet 3114, Bangladesh; c Atmospheric and Environmental Chemistry Laboratory, Atomic Energy Centre Dhaka 1000, Bangladesh; d Faculty of Fisheries and Food Science, Universiti Malaysia Terengganu, 21030 Kuala Nerus, Terengganu, Malaysia

**Keywords:** Trace metal, Environmental contamination, Spawning ground, Meghna estuary, Bangladesh

## Abstract

Meghna River Estuary, the largest estuarine system (GBM, Ganges-Brahmaputra-Meghna) in Bangladesh, is a major spawning ground of national fish, Hilsha shad. In this study, we collected 24 surface sediment and 24 water samples from the entire lower estuary (4 sites, 3 sampling points from each site, 2 replicas from each sampling point) to detect trace/heavy metals. Sediment samples were collected from the top surface soil (0–5 cm) using Ekman grab sampler and water samples from 5 cm below the surface layer using plastic water bottles. After collection, sediment and water samples were preserved as necessary using HNO_3_ (for water). Immediately after reaching the laboratory, sediment samples were dried in an oven at 70°C until the constant weight gained. The metals were then analyzed using energy-dispersive X-ray fluorescence method (EDXRF) and calculated the metal concentrations. In total, 12 metals were detected and the average value (mg/Kg) of all metals for sediment samples followed the descending order of Fe > Ca > *K* >Ti >Sr >Zr >Rb> Cu > Zn >Pb >As > Ni, and for water the order (µg/mL) of Fe >Ti > Ca > Co >Mn > Ni > Zn >Sr > Cu > As > Se . Besides, several physicochemical parameters i.e. water pH, soil pH, temperature, salinity, dissolved oxygen, hardness, and alkalinity of the 12 sampling points were also measured *in-situ* using handheld instruments.

**Specifications table**

**Table:** 

Subject	Pollution			
Specific subject area	Trace/Heavy metal contamination			
Type of data	Tables, figures			
How data were acquired	Physico-chemical parameters (e.g., pH, salinity) were measured by digital hand-held multi-meter (HANNA instruments, Germany). For metal analysis, 24 sediment and 24 water samples were collected from selected sampling points along the lower estuary using Ekman grab and plastic water bottles respectively during day time. Trace/ heavy metal data were measured by following standard procedure of sample preparation and analysis by the EDXRF (energy-dispersive x-ray fluorescence) method [1,2].			
Data format	Raw and Analyzed			
Parameters for data collection	1. Surface sediment samples from the bottom of the lower estuary of Meghna river. 2. Sub-surface water samples from the same area of sediment sample.			
Description of data collection	The sedimet and water samples were collected during June and July 2017 from 4 sites and 12 stations in the lower part of the Meghna estuary, Bay of Bengal (BOB), Bangladesh. Sediment and water samples were collected using Ekman grab and water bottles respectively. After collection, samples were carried to the laboratory for further analysis. Sediment samples were dried in an oven at 70°C until the constant weight. In the next step, we made homogeneous mixture of the sample and prepared 1 g weighed pellet using 10-ton pressure. The metals were then analyzed using energy-dispersive x-ray fluorescence method (EDXRF) and calculated the metal concentration value. In terms of water sample analysis, we followed the same process but we used cellulose for making a pellet. It is noted that EDXRF is a solid matrix sample analyzer and cellulose won't cause any change the metal concentration in the water sample. Before adding 1 g cellulose in 500 mL sample water, we filtered water samples by 0.45 µm membrane filter paper. Then, water was evaporated with a water bath and further dried at 70 °C in the IR lamp until constant weight. Besides, the rest of the process is the same as the sediment sample analysis.			
Data source location	Institution: Noakhali Science and Technology University City/Town/Region:Noakhali Country: Bangladesh			
	Latitude and longitude (and GPS coordinates, if possible) for collected samples/data:			
	**Station name**	**Location**	**Latitude(N)**	**Longitude(E)**
	Chandpur	S1	23.234036°	90.645845°
		S2	23.247469°	90.660404°
		S3	23.332988°	90.649363°
	Bhola	S4	22.26272°	90.9629°
		S5	22.25762°	90.9616°
		S6	22.32522°	90.98037°
	Hatiya	S7	22.15612°	91.04126°
		S8	22.15525°	91.04702°
		S9	22.36653°	91.12356°
	Sandwip	S10	22.49015°	91.43216°
		S11	22.5052°	91.54559°
		S12	22.50567°	91.5435°
Data accessibility	With this article			

**Value of the data**

• The data presents a comprehensive information on the levels and distribution of trace/heavy metals in both sediments and water of the largest estuary in Bangladesh. World scientific community can get reliable data on this subject from GBM estuarine system.
• Ecological and human health risks from accumulation of metals can be assessed from this data. Moreover, local fish farmers can use the data to avoid polluted sites for aquaculture.
• The data can be used as benchmark for future research. Environmentalists/ policy makers can use the information to control metal pollution in this valuable ecosystem.

## 1 Data description

The physico-chemical parameters i.e. pH, temperature, DO (dissolved oxygen), salinity, hardness and alkalinity were measured, and their average values are presented in Table 1. The mean ± SD values of water pH, soil pH, temperature, salinity, DO, hardness and alkalinity of the 12 sampling points were 7.12±0.27, 6.33±0.41, 27.97±2.94 °C, 0.14±.10 PSU, 7.83±1.89 mg/L, 204.67±98.28 ppm and 83.75±21.76 ppm, respectively (Table 1). All the values of measured parameters were within the acceptable range for fisheries and aquaculture activities. From elemental analysis, 12 trace/heavy metals were detected in water and sediment samples from the study area by the EDXRF method. In the case of sediment, the average concentration (mg/Kg) of detected metals followed the descending order of Fe (23,961.50) > Ca (16,984.17) > *K* (16,750.83) >Ti (2745.37) >Sr (190.42) >Zr (152.24) >Rb (131.45> Cu (38.10) > Zn (34.67) >Pb (9.81) >As (<4.17) > Ni (<0.17) (Table 2), whereas for water the mean levels (µg/mL) followed the order of Fe (188.13) >Ti (150.06) > Ca (108.06) > Co (41.32) >Mn (17.63) > Ni (7.51) > Zn (6.90) >Sr (5.93) > Cu (5.24) > As (4.39) > Se (4.24) (Table 3).

**Table 1:** Water quality parameters of Meghnar estuarine system.

Sites	Water pH	Soil pH	Temp. (ᵒC)	Salinity (ppt)	Hardness (mg/L)	DO (mg/L)	Alkalinity(ppm)
S1	6.9	6.7	24.5	0.1	100	5.0	70
S2	6.9	6.5	24	0.1	140	5.2	73
S3	6.6	6.6	24	0.1	120	5.0	67
S4	7.4	5.8	28.7	0.1	180	8.5	82
S5	7.4	5.8	28	0.0	190	8.4	68
S6	7.4	5.9	28	0.0	160	7.5	83
S7	7.4	6.2	28.5	0.2	210	9.5	69
S8	7.3	6.2	28	0.2	420	8.8	71
S9	7.1	6.1	28	0.1	410	9	74
S10	6.7	6.6	32	0.2	270	9.6	126
S11	7.2	6.9	32	0.3	130	8.4	114
S12	7.1	6.7	30	0.3	126	9.1	108
Mean ± SD	7.117 ± 0.269	6.333 ± 0.412	27.974 ± 2.937	0.142 ± 0.099	204.668 ± 98.281	7.833 ± 1.894	83.75 ± 21.757

**Table 2:** Metal concentrations (Mean±SD) in water of Meghna estuarine systems in 2017.

Metal (µg/mL)	Sampling stations				Average
	Chandpur S1-S3	Bhola S4- S6	Hatiya S7-S9	Sandwip S10-S12	
Ca	54.76 ± 27.59	168.19±21.17	114.85±37.08	94.44±38.82	108.06±47.22
Ti	102.08±49.14	160.88±44.57	146.99±11..31	19.28±61.84	150.06±36.72
Mn	13.52±0.44	21.99±5.80	15.08±1.76	19.98±4.90	17.63±3.10
Fe	23.48±10.15	315.62±105.45	175.11±20.04	238.31±128.85	188.13±123.90
Co	42.97±33.67	20.60±5.56	13.94±0.73	87.76±66.24	41.32±33.36
Ni	7.68±0.67	7.02±0.47	7.26±0.23	8.08±1.61	7.51±0.47
Cu	5.21±0.23	4.86±0.14	4.93±0.46	5.94±0.79	5.24±0.40
Zn	10.30±5.86	6.18±0.44	6.06±0.88	5.06±1.68	6.90±2.32
As	4.59±0.52	4.40±0.29	4.26±0.12	4.31±0.27	4.39±0.15
Se	4.37±0.13	4.04±0.25	4.71±0.79	3.88±0.36	4.25±0.37
Sr	2.16±0.56	8.22±2.73	4.17±0.34	9.17±2.65	5.93±3.32
Ca	54.76 ± 27.59	168.19±21.17	114.85±37.08	94.44±38.82	108.06±47.22
Ti	102.08±49.14	160.88±44.57	146.99±11..31	19.28±61.84	150.06±36.72
Mn	13.52±0.44	21.99±5.80	15.08±1.76	19.98±4.90	17.63±3.10
Fe	23.48±10.15	315.62±105.45	175.11±20.04	238.31±128.85	188.13±123.90
Co	42.97±33.67	20.60±5.56	13.94±0.73	87.76±66.24	41.32±33.36
Ni	7.68±0.67	7.02±0.47	7.26±0.23	8.08±1.61	7.51±0.47
Cu	5.21±0.23	4.86±0.14	4.93±0.46	5.94±0.79	5.24±0.40
Zn	10.30±5.86	6.18±0.44	6.06±0.88	5.06±1.68	6.90±2.32
As	4.59±0.52	4.40±0.29	4.26±0.12	4.31±0.27	4.39±0.15
Se	4.37±0.13	4.04±0.25	4.71±0.79	3.88±0.36	4.25±0.37
Sr	2.16±0.56	8.22±2.73	4.17±0.34	9.17±2.65	5.93±3.32

**Table 3:** Elemental concentrations (Mean±SD) in sediment of Meghna estuarine system in 2017.

Metal (mg/Kg)	Sampling stations				Average
	Chandpur	Bhola	Hatiya	Sandwip	
	S1-S3	S4-S6	S7-S9	S10-S12	
K	13,833.33±1572.59	19,326.67±1797.23	19,260.00±5253.88	14,583.33±1111.32	16,750.83±2951.88
Ca	17,396.67±926.14	17,840.00±3960.50	15,610.00±2597.61	17,090.00±2054.82	16,984.17±966.46
Ti	2950.25±59.79	2684.88±316.72	2844.10±399.13	2502.25±169.00	2745.37±195.35
Rb	117.31±8.78	131.96±7.92	145.73±20.38	130.82±21.23	131.45±11.61
Fe	23,140.00±831.62	23,190.00±163.71	25,770.00±3260.49	23,746.67±2328.01	23,961.50±1236.55
Pb	12.41±6.89	7.65±5.55	9.65±7.37	9.52±2.03	9.81±1.96
Cu	38.82±1.54	39.21±2.52	37.81±5.57	36.57±2.78	38.10±1.18
Zn	33.43±5.08	33.24±1.94	36.89±2.80	35.02±1.35	34.64±1.70
Zr	153.29±19.41	152.43±6.21	156.78±9.67	146.45±25.02	152.24±4.29
Sr	208.94±22.63	163.60±13.00	202.12±8.40	187.01±31.00	190.42±20.02

## 2 Experimental design, materials, and methods

### 2.1 Study sites

**Fig. 1:**
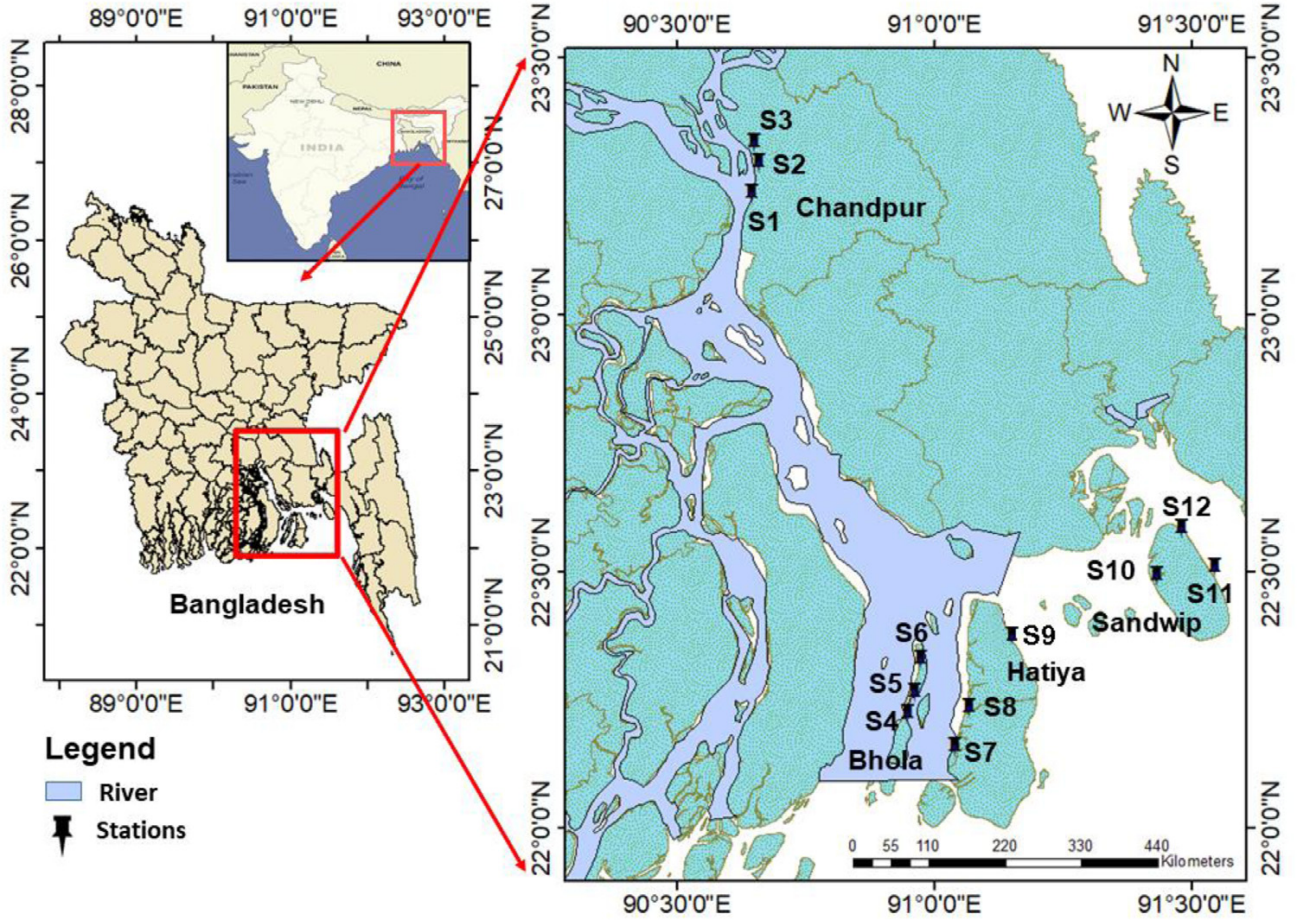
Location of the lower Meghna river estuarine system showing sampling stations (S1-S12), Bay of Bengal, Bangladesh.

The estuary of the lower part of Meghna River is an important area for being the Hilsha (*Tenualosa ilisha,* the national fish of Bangladesh) spawning ground along the Bay of Bengal (BOB) coast line. It is the largest estuarine system in Bangladesh where the three mighty rivers, Ganga, Brahmmaputra and Meghna are coincided. We selected four sampling sites (Chandpur, Bhola, Hatiya, and Sandwip) from up to down stream region of the lower estuarine system and three sampling points from each sites (Fig 1). 48 samples (24 water and 24 sediment) were collected from the 12 selected stations. Furthermore, the upstream site was Chandpur and then following the downstream sites i.e. Bhola, Hatiya, and Sandwip. We have tried to maintain at least 1.5 km distance from one point to another within each site. All the sampling points were recorded by digital GPS (Global Positioning System) meter and a sampling map was generated by Arc GIS software.

### 2.2 *In-situ* water quality parameters measurement

During sampling of water and sediment, we recorded seven physico-chemical parameters i.e. water pH, soil pH, temperature, salinity, alkalinity, dissolved oxygen, hardness from the all sampling points *insitu* by different portable digital meters (HANNA, Germany). Firstly, 200- 300 ml of water were collected in a glass beaker on site, and then the logger of digital meters were inserted into the water to record the values of each parameter.

### 2.3 Water and sediment sample collection

In total, 24 water samples were collected by immersing plastic bottles from 12 stations in the study area during June and July 2017. Water samples were collected from below the 5 cm of the surface water for avoiding surfactants. Besides, plastic bottles (polyethylene) were used to store the sample using 5% of nitric acid, HNO_3_ (pH= 2). Before storing the sampled water, plastic bottles were washed thoroughly by distilled water and then rinsed with sampling water. Later on, samples were transported to the laboratory with an ice-box for further analysis.

In the case of sediment sampling, 24 samples were collected by Ekman grab sampler (Seabird Company, USA) from the bottom surface (0 to 5 cm) of the sampling stations. Immediately after that, we kept our sample in polyethylene bags and transported it to the chemistry lab at Atomic Energy Centre, Dhaka for further analysis.

### 2.4 Sample analysis

In the case of water sample analysis, we took 500 mL of each water sample in a porcelain dish. Then the taken sample was filtered by 0.45 µm membrane filter paper (Sigma-Aldrich, USA). After that, adding 1 g of cellulose powder and the samples were evaporated in the water bath and then further dried under an IR lamp at about  70°C till constant weight was attained. Next, the residue was ground and prepared powder using mortar and pestle. Then we made a 1 g weighed pellet by applying hydraulic pressure of approximately 3 tons for 2–5 min using a pellet maker (Specac, UK). In the next step, the pellet was kept into the XRF system at a voltage of 25 V and a current of 50 μA in an X-ray tube for approximately 18 min. For determining the characteristics of the X-ray, Si–Li detector system was used in a solid-state. Finally, for checking the accuracy and precision of the EDXRF analysis, a blank and certified reference material (Marine sediment, IAEA 433) were run by using the same procedure that was followed for the experimental samples. EDXRF is a good method for detecting metal in a solid matrix that's why we used IAEA 433 as reference material for both samples. Finally, we got the metal concentration result after calculation of the peak area by ADMCA and FP-CROSS program as spectrum analysis.

In terms of sediment samples, collected samples were allowed to settle for removing excess water. All samples were then taken into porcelain dishes separately. Each dish with a particular sample was placed in an oven at around 70°C until a constant weight was obtained. The dried mass of each sample was then pulverized to a fine powder using a mortar and pestle, and preserved in a plastic vial with the identification mark inside desiccators. After that, the homogeneous powder was used to prepare a 1 g weighed pellet using 10-ton pressure by a pellet maker (Specac, UK) for elemental analysis by EDXRF. In the next step, we placed the prepared pellet on the XRF system and got the concentration of the metal in the same way. The detailed of the aforementioned EDXRF method is found elsewhere[1, 2]. Besides, most of the researchers’ from all over the world have chosen this method for heavy metal detection from sediment and water samples like Hossain et al. [2].

## Declaration of Competing Interest

There is no competing interest.

## Appendix Supplementary materials

Image, application 1
